# Professional Training of Future Preschool Teachers in the Field of Artistic and Aesthetic Education by Means of Contextual Learning Technologies

**DOI:** 10.3390/bs10020050

**Published:** 2020-02-04

**Authors:** Olha Krasovska, Nataliya Miskova, Alla Veremchuk

**Affiliations:** 1 Department of Primary and Pre-school Education, International University of Economics and Humanities Named after Academician Stepan Demianchuk, 33028 Rivne, Ukraine; n.miskova@i.ua; 2 Department of Pedagogy of Primary Education, Rivne State Humanitarian University, 33000 Rivne, Ukraine; allavera99@ukr.net

**Keywords:** professional training, future preschool teachers, contextual learning technologies

## Abstract

The article deals with the development peculiarities of the subject artistic competence of future preschool teachers in the field of artistic and aesthetic education of children by means of contextual educational technologies. The students in question were being observed during the classes of Fundamentals of the Fine Arts with Methodology, the lessons of Decorative Arts with Methodology, as well as the Artistic Production and Design Fundamentals sessions. The purpose of the article is to reveal the methodology of contextual learning technologies’ implementation into the process of future preschool educators training and check their effectiveness in the realm of children’s artistic and aesthetic education experimentally. In the course of the research, we used such methods as analysis and synthesis of psychological, pedagogical, and art sources, as well as studying and generalization of the current state of future preschool teachers professional training in the field of artistic and aesthetic education. We also employed analysis, comparison, and classification with the aim of determining the essential characteristics, criteria, and levels of future preschool teachers’ subject competence in artistic and aesthetic education. Another approach that we turned to was that of pedagogical experiment with the further qualitative and quantitative analysis of its results using the Kolmogorov–Smirnov statistical criterion. The outcomes of the experiment brought about a need for the implementation of contextual learning technologies into the development of future preschool educators’ subject competence.

## 1. Introduction

What are contextual learning technologies? Technologies of contextual learning comprise the system of didactic forms, methods, and tools that simulate the substantive and social content of the future professional activities of the specialist [1]. At the same time, the acquisition of knowledge and the development of competences are at the core of these activities. The main task of contextual learning technologies is to ensure the proactive nature of the individual’s activities, which contribute to the formation of the necessary subject-professional and social qualities of the specialist.

Contextual technologies of education make it possible to create the conditions for the interpenetration between academic and future professional activities as one of the ways to achieve professional competence. The objective of the said technologies is to implement the educational process into the context of future professional activities by means of introducing actual links and relations into various forms and methods of education, provided by higher educational establishments, as well as solving specific professional tasks requiring the formation of a number of special competences, as argued by Verbytskyi [1]. According to such scholars as Clelland and Mansfild [2], Raven, Hoffman and Linard [3], Silver [4], and Slavin [5], competence education is at the heart of the process of a future teacher training. 

By contextual learning we mean the development of such a model of the educational process, which forms the subject and social content of the professional activity, providing the conditions for the transformation of the student’s educational activities into the professional activities of a specialist, as formulated by Bulanova-Toporkova [6]. Contextual technologies in the professional training of a teacher are characterized by aspects such as transferring the emphasis from the teaching activity of the university professor to the student’s cognitive performance, his work, and activity.

Another aspect is the bilateral interaction in the “teacher–student” system on the basis of mutual understanding, openness, trust, stimulation, and support of cognitive creativity, as well as the process of formation of the necessary professional traits and qualities. Other conditions that contribute to the success of this interaction are providing psychological and pedagogical conditions, forms and methods of educational activities that assist in the shaping of the professional competences, general and professional abilities, social qualities of the individual, and the process of gaining experience in creative activity.

Skvortsova [7] suggests that context learning is a form of implementation of a dynamic model of student activities: from their own learning activities (for example in the form of lectures) through quasi-professional activities (game forms of studying, special courses) and vocational-professional activities (research work of students: term papers and thesis, pedagogical practice, etc.) to their own professional activities. The main parameter of the educational process of contextual type is the modeling of the subject and social content of future professional activity through the reproduction of real professional situations. The basic forms of contextual education are as follows: learning activities of academic type (lectures, seminars, practical sessions, laboratory classes, individual work); quasi-professional activities (business games, game forms of studying); and educational-professional activities (research work, industrial practice). Among the forms that are transitional from one basic type to another there are laboratory and practical classes; simulation modeling; analysis of situations of professional activity; role-plays; and special courses and seminars. 

Modern scientific–pedagogical research reveal various aspects of the professional training of 012 “Preschool education” students, but the problem of forming future preschool teachers’ subject competence in the artistic and aesthetic education of children by means of contextual education technologies has not yet been thoroughly investigated [1,6,7]. Subject artistic competence of the future preschool teachers is the ability to apply professionally-oriented artistic knowledge, skills, and competences that constitute the theoretical and activity-technological basis of the educational field entitled “A child in the world of culture” as a part of the core component of preschool education in general and its separate elements in particular. There are various components of the subject artistic competence of the future preschool teachers, among which there are musical, visual, artistic, and synthetic competences.

The purpose of the study is to reveal the peculiarities of the implementation of context-based learning technologies in the process of training future preschool teachers in the field of artistic and aesthetic education during the Fundamentals of Fine Arts with Methodology classes, the lessons of Decorative Arts with Methodology as well as the Artistic Production and Design Fundamentals sessions.

## 2. Materials and Methods

The study subjects were 20–22-year-old students at their 3rd, 4th, 5th, and 6th year of studying the specialty 012 “Preschool Education”. In total, 606 students were involved in the experiment, with 298 and 308 of them in the experimental and the control groups, respectively. The authors implemented a complex of contextual technologies of education in the professional training of the experimental group students, which hypothetically had to generate an increase in qualitative and quantitative indicators of the future preschool teachers’ subject competence in the artistic and aesthetic education of children. Professional training of students in the control group was carried out according to traditional methods of teaching in higher education institutions. It was mostly represented by the lecture-seminar educational system, which includes lectures, seminars, practical classes, and different forms of independent, individual, and research work of students. All participants had given their informed consent for inclusion before participating in the study. Furthermore, the study was conducted in accordance with the Declaration of Helsinki on the premises of the Ethical Committee of the Primary and Pre-school Education of the university.

To solve the given tasks, the following investigation methods were used: theoretical—scientific analysis and synthesis of psychological, pedagogical, and artistic sources, as well as Internet resources; empirical—tests, interview, questionnaire and expert marking, and a pedagogical experiment with a qualitative and quantitative analysis of the results. Data integrity was checked using the Kolmogorov–Smirnov statistical criterion and using the linear correlation method of K. Pearson. Apart from that, the method of E. Pustylnic was utilized for checking the accordance of empirical data to the laws of normal distribution and factor analysis (with the help of pack SPSS Statistics 17.0 software).

## 3. Results

### 3.1. Pedagogical Conditions and Stages of the Experiment

The pedagogical conditions of the experimental methodology included a system of measures:

- Creation of an innovative environment for professional preparation of “Preschool education” students for artistic and aesthetic activity in the conditions close to the future professional-pedagogical activity;
- Introduction of organizational and methodological innovations for the development of isolated components of the subject artistic competence of a future preschool teacher. The said innovation encompassed various printed and video materials, along with some educational computer programs, namely electronic and multimedia manuals, reference and information systems, training programs for knowledge consolidation, and monitoring programs aimed at checking the completed work;
- Innovative and pedagogical orientation of the content of methodological disciplines and art disciplines in higher education institution with the inclusion of contextual pedagogical technologies in teaching the disciplines of Fine Arts with Methodology, Teaching Decorative Art with Methodology, and Artistic Production with Design Fundamentals.

The implementation of contextual education technologies is executed in three stages: informational–operational, activity-technological, and reflexive-creative. The program of the pedagogical experiment included three interrelated stages, which provided for the consistent implementation of the educational program for students of the specialty 012 “Preschool education”: the 2014–2015 academic year (adaptation-information stage); the 2016–2017 academic year (activity and technological stage); and the 2018–2019 academic year (reflexive-creative stage). Each of the stages incorporated the developed examining tasks with the defined contents. We have refined and expanded the content of each stage, and defined the pedagogical conditions for the formation of the subjective artistic competence of future preschool teachers.

### 3.2. The Purpose of the First Informational–Operational Stage (Third Year of Study)

The purpose of the first informational–operational stage (third year of study) was to give future preschool teachers a notion about their future profession, the specifics of artistic and aesthetic education of preschool children, the peculiarities of the system of psychological, pedagogical, methodological, and artistic knowledge, practical artistic and creative competencies, and mastering of artistic techniques.

The main condition for the successful implementation of the informational–operational stage is the creation of an innovative environment for the preschool educators’ professional training in the field of artistic and aesthetic education, aimed at self-realization of a future specialist with the help of contextual education technologies. At this stage in the practice of teaching Fundamentals of Fine Arts with the Methodology of Management the following pedagogical technologies became of use: a problem solving lecture and practical lesson (proper educational activity), during which the objective context of professional activities is formed; practical training with elements of discussion, role-plays and simulation games, and future professional activities modeling (quasi-professional activity), which contribute to the formation of both the subject and social context of future professional activities; students’ research work, project preparation, pedagogical practice, and term paper completion (educational and professional activity). 

During the practical lesson on methods of teaching fine arts, students were encouraged to participate in the discussion under the title “The role of traditional and modern interactive methods and techniques of teaching fine arts”, as well as asked to prove the expediency of the research, heuristic, and problem statement teaching methods for enhancing creativity in children. Apart from that, the students had to develop a task of research, heuristic, and problematic nature to help children master such fundamental aspects of artistic literacy as color, form, composition, volume, and space. In the course of the preparation to the aforementioned activities, the students needed to work independently on different educational and methodological literature, in order to make a comparative description of these methods and substantiate the most reasonable point of view; they were also asked to illustrate the certain methodology by means of solving specific methodological tasks. Such conditions are beneficial for the formation of future professional activities’ subject context.

In order for students to get acquainted with the alternative pedagogical technologies of teaching art disciplines in elementary school, we suggest considering a series of tasks that involve using these technologies. In particular, students need to prepare reports and presentations on the following topics: “Modern artistic education in Great Britain, the Netherlands, and France”, “Artistic and aesthetic education in Japan”, “Teaching of artistic disciplines in the Waldorf education system”, “Art in Maria Montessori’s system of education and upbringing”, etc. To discuss these presentations, we arrange group and intergroup discussions. Discussions are considered successful if students receive questions in advance, which allows them to use up-to-date data and facts, hence make further communication more efficient. 

Defending the chosen stance with the help of imitation technologies is implemented during the practical sessions, where the students have the opportunity to illustrate their viewpoint by presenting a fragment of a lesson on developing a certain artistic skill. Such sessions are practice-oriented—not only do the students reproduce the knowledge gained during the lectures, but they also express their own position and model the activities of both educator and children in potential real-life situations; they carry out an analysis of specific methodological situations and assess them from the point of view of teachers and methodologists. It must be noted that the way in which the modeled situation unfolds is in no way predetermined and depends mostly on the actions of the “teacher” and the “children”; thus, it allows for the formation of both the subject and social context of future professional activities. 

### 3.3. The Purpose of the Second Activity-Technological Stage (Fourth Year of Study)

The purpose of the second activity-technological stage (fourth year of study) is the formation of future preschool educators’ ability to implement the functions of artistic and aesthetic education in actual preschool teaching, to master general pedagogical and specific artistic technologies, and to practice using non-standard creative techniques. 

To achieve this goal, an optimal combination of the theoretical and practical components of the 012 “Preschool education” students’ preparation for artistic and aesthetic education was developed, making possible the acquisition of general and professional competences (pedagogical, psychological, methodical, and artistic ones) during the classes of Fine Arts with Methodology of Teaching and Artistic Production and Design Fundamentals. In order to provide the conditions for reaching the activity-technological stage goal, we created the necessary artistic learning context, optimized students’ independent and research work, and developed the technologies of contextual education that involve intellectual team activities, such as producing common ideas, holding cognitive discussion, and modeling various processes (simulation exercises, analysis of professional situations, and trainings). Imitation technologies include role-sharing, situation modeling, and business games, which are divided into operational-role-based, problem-oriented, educational-role-based, and educational-pedagogical ones. For instance, educational-pedagogical games were used to choose the best ways for teaching students the methods and techniques of real-life pedagogical activities in the preschool institutions; they also required that students knew specific topics, along with the foundations of pedagogy, psychology, and methodology of teaching Fine Arts.

Practical and laboratory classes on “Decorative Art with Methodology of Teaching”, “Teaching and Artistic Production”, and “Design Fundamentals” were conducted with the help of such technologies of contextual learning as imitation games, round table discussions, seminars, group discussions, case studies, project development, group work, writing essays, and pedagogical trainings. These technologies presupposed that students were modeling some typical vocational-pedagogical situations in the field of art education that take place in the preschool environment, while individual and group trainings in the classroom made it possible to develop professionally important qualities and to improve students’ methodological and artistic skills. The introduction of contextual technologies to the preparation of future preschool educators involves not only teaching music and fine arts, but also inviting well-known artists and folk craftsmen as partners to the teachers.

For instance, we held a master class in the production of traditional folk children’s linen toys with craftswoman Victoriia Kisilenko. After that, painters Anatoly Ivanenko and Ruslan Hves taught our students some hands-on techniques of painting and held casual discussions on the possible connotations of paintings by such Ukrainian artists as Ivan Trush, Heorhii Narbut, Kazimir Malevich, Adalbert Erdelish, and Mykola Glushchenko during the Euro-Art Gallery exhibition. 

Among the tasks of the contextual nature we offered the following ones:

1. Preparation of a class plan with elements of game simulation of the artistic and aesthetic cycle.
2. Preparation of a class plan, including art-therapy and suggestopaedia in the artistic and aesthetic disciplines.
3. Preparation of a class plan with the elements of integrated teaching of music and the arts.
4. Preparation and presentation of an artistic project on the formation of the culture of understanding art by children.
5. Preparation of computer-assisted presentations, open teaching forms, interactive video materials, texts, and multimedia packages.

### 3.4. The Aim of the Third Reflexive-Creative Stage (Fifth Year of Study)

The aim of the third reflexive-creative stage (fifth year of study) is to form the future educator’s ability to reflect on their own professional experience and to improve their skills in artistic and aesthetic education in the conditions of the preschool education institution. 

This goal has been achieved by the stabilization and correction of the already formed psychological, pedagogical, methodological, and artistic knowledge and skills of 012 “Preschool education” students, by developing an individual style of professional activity in the field of artistic and aesthetic education, as well as the ability of self-assessment and assessment of the gained experience; and by consolidating skills and abilities of independent research work. Conditions for realizing the goal of the reflexive-creative stage are provided by the means of developing a special professional course “Artwork and the Basics of Design” that involves contextual technologies, such as artistic project activities, master classes on the basics of folk art, pedagogical experience exchange, “Creative Workshop of Students’ Artistic Education”, and a pedagogical portfolio of the preschool teacher.

At this stage, a series of laboratory sessions in the form of master classes on folk decorative and applied arts were organized based on the use of productive and creative technologies of fine arts. The topics of the mentioned master classes covered the areas of applied arts and handcrafts, namely “Easter egg decoration technology”, “The technology of making a vytynanka”, “The technology of developing a stained glass window sketch”, and “The technology of Petrykivka painting”. 

During the laboratory-practical course of the artistic and pedagogical training of future preschool educators, special classes and creative workshops for mastering the alternative technologies of Waldorf pedagogy were conducted, which regarded the following aspects:

Artistic and creative activities in the Waldorf pedagogical system on the basis of the reproduction of fairy tale images in the watercolor painting technique “wet in wet”; drawing the fairy tale episodes with wax chalk; painting with a focus on the nature of the colors;

Clay modeling as a way for developing a sense of shape; methodology of modeling an animal from the entire clay piece, representing gestures and movements; wax modeling as a means of developing fine motor skills;

Creation of black and white graphic images; playing the game “a dialogue with the artist” on the basis of Vincent Van Gogh’s art;

Experiencing modern art history; studying impressionists and expressionists art: transforming the classical image of an apple into an impressionistic one, reflecting emotions in painting.

In the future educators’ professional preparation for the artistic and aesthetic education of children, we have highlighted the need of forming methodological and artistic ideas and knowledge, along with studying the students’ values in the field of artistic culture. Pedagogical essays, written by the students, helped to reveal some interesting opinions.

Svitlana O.: “Art education is aimed at forming a special culture of perception of the world around us, a culture of development of personality’s abilities to transform themselves and the surrounding reality. Art teaches people to see the world in the diversity of its forms, notions, and colors, shapes their worldview and attitudes. As a future educator, I will focus on the moral and aesthetic development of children, and my pedagogical credo will be: ‘Art is a means of harmonizing a child’s personality and an important educational factor’”. 

Halyna S.: “Modern children prefer material values. During the time of my internship, I realized that working with such children would be a tall order, as we would have to explain to them that there is more to life than its material plane. However, children are sensitive to and capable of creating art. I think that art education is a powerful impact factor in the formation of their spirituality”. 

Ivanna K.: “Art teaches people to see the world in the diversity of its forms, notions, and colors, and shapes their worldview and attitudes. Implementation of artistic disciplines at all levels of education enables children to realize their life purpose, form aesthetic and moral feelings, and develop their creative abilities”.

Modern methodological approaches to providing proper training for future preschool education specialists require a reorientation to an individually differentiated, personally oriented model of learning; introduction of methods and means of checking students’ knowledge; and organization of self-education activities. For this purpose, interactive methodical complexes on different disciplines have been developed, which, according to modern requirements, are given to students electronically on a CD, or in the form of an Internet page in the university e-library. The structure of each complex includes various educational materials, such as printed hand-outs, video materials, and computer training programs—that is, traditional elements; and informational-methodical resources—electronic guidelines, multimedia manuals, reference systems, training programs for the consolidation of knowledge, and control programs for its checking. To develop such programs, we used Microsoft Word packages, Excel, Microsoft Power Point, Corel Draw, Open Office, orgImpress, Teach Book Constructor, etc.

**Figure 1:**
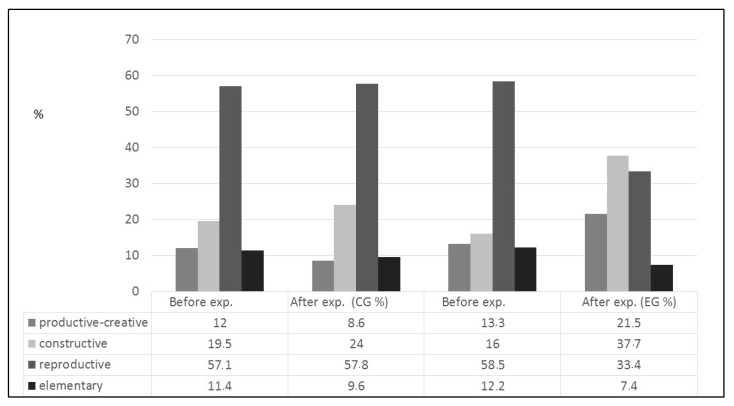
Distribution of respondents by levels of formation of the subject artistic competence.

At the end of the experiment, we were able to trace the positive dynamics of an increase in the level of the subject competence among the students of the experiment group, while the control group presented less significant changes. The results of the final control (Table 1, Figure 1) showed that in the experiment group, where intensive educational technologies were implemented, there occurred some statistically significant shifts towards the growth of the future primary school teachers’ professional readiness for the artistic and educational activities in general.

## 4. Discussion and Conclusions

The positive dynamics in the formation of future educators’ subject competence in the field of artistic and aesthetic education testifies to the effectiveness of the implementation of contextual technologies. This is further confirmed by the results of the quantitative and qualitative analysis, conducted with the help of Kolmogorov–Smirnov’s statistical criterion. We believe that the process of training future educators in preschool establishments can improve, if such contextual technologies as modeling, simulation games, master classes, and creative workshops are consistently implemented in their studying.

Our research allowed us to obtain answers to the following questions. Is the strategy of contextual education technologies effective in the process of forming the subject competence of future preschool educators in the artistic and aesthetic education of children? What types of contextual technologies are most efficient in the professional training of future teacher? Will the implementation of context technologies in the process of professional training of 012 “Preschool education” students lead to an increase in qualitative indicators of the subject competence of future preschool educators in the artistic and aesthetic education of children?

Hence, we proved that a strategy of contextual education technologies is effective in the process of forming the subject competence of future preschool educators in artistic and aesthetic education of children. Contextual technologies of education make it possible to create the conditions for the interpenetration between academic and future professional activities as one of the ways to achieve professional competence.

Types of contextual technologies that are most efficient in the professional training of future teachers are: laboratory and practical classes; simulation modeling; analysis of situations of professional activity; role-plays; special courses; and seminars. The basic forms of contextual education are as follows: learning activities of the academic type (lectures, seminars, practical sessions, laboratory classes, individual work); quasi-professional activities (business games, game forms of studying); and educational-professional activities (research work, industrial practice).

The implementation of context technologies in the process of professional training of 012 “Preschool education” students lead to an increase in qualitative indicators of the subject competence of future preschool educators in the artistic and aesthetic education of children.

According to the results of the experimental activities that we have carried out on the formation stage, a considerable part of the future preschool educators from the experimental group demonstrated a constructive, productive, and creative level of the subject artistic competences, striving for the quality fulfillment of the professional tasks, orientation for pedagogical cooperation, and the ability to argue their own opinions with the aim of achieving the goals of the artistic and aesthetic activities. With such a level of diligence and motivation, a person who possesses already defined artistic interests is oriented on cultural values, and perseveres in achieving the goals of the pedagogical activities.

The outcomes of the experimental work suggest positive dynamics in the constructive, productive, and creative levels of students from the experimental group. At the productive, creative, and constructive level, the students have the skills of organizing activities connected to arts and education and the ability to use the basic forms and methods of these activities in the context of the preschool education; they also can integrate the knowledge on different artistic subjects in modeling of the artistic and educational activities and reach the goals, tasks, and functions of such a type of pedagogical work. Modernization of the professional preparation of the future educators by introducing the experiment on the basis of contextual educational technologies has become an essential condition in the formation of the thematic artistic competence of future preschool educators, their ability to be professionally creative, flexible, and ready to solve any pedagogical issues. 

The positive dynamics of raising the level of professional training of the students of the experimental group was determined. The changes of the control group were less significant. The results of the tests were: 12% of the graduates had a productive-creative level of formation of professional training of the control group at the beginning of the experiment and 8.6% of the graduates after experiment. The indicators of the experimental group were 13.3% and 21.5%. 19.5% of the respondents had a constructive level of formation of professional training before the experiment and 24% of the graduates after it. Significantly higher results of the experimental group were shown (16% and 37.7%). Almost the same number of students had a reproductive level at the beginning and at the end of the experiment (57.1–57.8%). The number of students of the experimental group has changed significantly (58.3% and 33.4%). The elementary level of the control group changed from 11.4% to 9.6% of the total number of respondents. The number of students of the experimental group significantly decreased from 12.2% to 7.4%. This result was actually an increase. It consists 14.14% of the professional training to artistic-education activity. It was a positive result. Data integrity was checked using the statistical criterion of the Kolmogorov–Smirnov method and using the linear correlation method of K. Pearson and the method of E. Pustylnic for correspondence of empirical data to the laws of normal distribution and factor analysis (with the help of pack SPSS Statistics 17.0 software).

## Figures and Tables

**Table 1:** The dynamics of the formation of the subject artistic competence of future preschool educators before and after the experiment.

Levels	Control Group				Experiment Group			
	Before the Experiment (n = 308)		After the Experiment (n = 308)		Before the Experiment (m = 298)		After the Experiment (m = 298)	
	f	%	f	%	f	%	f	%
Productive-creative	37	12.0	26	8.6	40	13.3	64	21.5
Constructive	60	19.5	74	24.0	48	16.0	112	37.7
Reproductive	176	57.1	178	57.8	173	58.5	99	33.4
Elemental	35	11.4	30	9.6	37	12.2	23	7.4
